# Effect of sample treatment on the elastic modulus of locust cuticle obtained by nanoindentation

**DOI:** 10.3762/bjnano.13.33

**Published:** 2022-04-22

**Authors:** Chuchu Li, Stanislav N Gorb, Hamed Rajabi

**Affiliations:** 1 Functional Morphology and Biomechanics, Institute of Zoology, Kiel University, Kiel, Germany; 2 Division of Mechanical Engineering and Design, School of Engineering, London South Bank University, London, UK

**Keywords:** biomimetics, cuticle, locust, material properties, mechanical testing, nanoindentation, water content

## Abstract

Cuticle is one of the most abundant, but least studied, biological composites. As a result, it has contributed very little to the field of biomimetics. An important step to overcome this problem is to study cuticle biomechanics by means of accurate mechanical measurements. However, due to many reasons, mechanical testing on fresh cuticle specimens is not always possible. Hence, researchers often use stored specimens to measure properties of arthropod cuticle. Our knowledge about the influence of different treatment methods on cuticle properties is currently very limited. In this study, we investigated the effect of freezing, desiccation, and rehydration on the elastic modulus of the hind tibial cuticle of locusts obtained by nanoindentation. We found that all the mentioned treatments significantly influence cuticle properties. This is in contrast to previous reports suggesting that freezing did not significantly influence the elastic modulus of native cuticle specimens tested in bending. In the light of our data, we suggest that changes of the elastic modulus of cuticle are not solely due to changes of the water content. Our results provide a platform for more accurate measurements of cuticle properties.

## Introduction

Cuticle is a lightweight material that forms the whole exoskeleton of insects, from the flexible intersegmental membrane to the stiff jaws and claws. Cuticle of each insect body part has undergone adaptations that enable it to withstand loads and displacements that are unique to that body part. Such adaptations are achieved through changes in, inter alia, microstructure and sclerotization and have provided cuticle with one of the widest recorded ranges of the elastic modulus ever, that is, 1 kPa to 20 GPa [1].

Owing to developments in mechanical testing [2] and imaging techniques [3] and the use of evolutionary algorithms [4], our knowledge about the biomechanics of insect cuticle has been widely broadened recently. However, cuticle remains to be one of the least studied biological materials. This is mostly because measuring the mechanical properties of insect cuticle is very challenging in practice. One of these challenges is associated with the rather fast desiccation rate of cuticle, as it loses its water shortly after removal from insect body [5]. Only small changes in the water content can significantly influence cuticle properties and, thereby, the obtained results [6–8].

Mechanical testing of fresh cuticle samples, in contrast, is not always possible. For example, when insects are not locally available, or when they cannot be kept in a laboratory, insect specimens must be stored. Thus, it is necessary to know how sample treatment methods may affect the properties of cuticle. A recent study by Aberle et al. [9] tried to address this question by testing cuticle specimens that were stored using standard procedures, including maintaining samples in water, ethanol, and glutaraldehyde as well as freezing and desiccating them. The authors tested the differently treated samples by three-point-bending tests and found that all the treatments, except for freezing, significantly affect the elastic modulus of fresh locust tibial cuticle [9].

A recent study suggests that, in addition to the treatment procedure, cuticle properties can also be influenced by the testing method [10]. There are significant differences between the elastic moduli of cuticle specimens tested in bending, nanoindentation, tension, and uniaxial compression [10]. This raises the question whether results obtained by Aberle et al. [9] would still be valid if another testing method, such as nanoindentation, is used for measuring cuticle properties. To address this question, here we study the effect of different sample treatments, including freezing, desiccation, and rehydration, on the elastic modulus of the cuticle of desert locust, a well-established model species in studies of insect cuticle. We used nanoindentation as the testing method because it is one of the most widely used experimental approaches for measuring cuticle properties. Furthermore, unlike most macromechanical testing methods, the properties obtained from nanoindentations are not influenced by the geometry of specimens. Hence, nanoindentation is one of the most suitable methods to measure the mechanical properties of cuticle specimens, which usually have complicated shapes. We chose locust hind tibiae as they were used in the former study by Aberle et al. [9], enabling us to compare our results with those from the literature. Our results not only allowed us to systematically analyze the effect of different treatments on the mechanical properties of insect cuticle but also to provide a guideline for more accurate measurements of the mechanical properties of insect cuticle in the future.

## Materials and Methods

### Ethics

All procedures in this study comply with ethical guidelines at Kiel University.

### Sample preparation and treatment

The desert locusts *Schistocerca gregaria* were obtained from pet shops in Kiel, Germany. They were raised under controlled temperature (25–30 °C) and humidity (30%–40%), kept under natural day/night light and fed with fresh vegetables. Prior to any experiment, insects were euthanized with CO_2_. We used only adult individuals, 21 days after imaginal molt. Hind leg tibiae were cut off directly below the femur–tibia joint with sharp razors and subdivided into four groups. Specimens in each group were subjected to one of the following treatments. (i) Fresh treatment: The samples in this group were used in their fresh state quickly after being cut from the body (less than 2 min) without any additional treatment. (ii) Freezing treatment: Fresh hind tibiae were stored in 0.5 mL Eppendorf tubes (Carl Roth GmbH & Co. KG, Karlsruhe, Germany) and kept at −20 °C for 48 h without medium. Frozen samples were thawed for 15 min in distilled water to room temperature. (iii) Desiccation treatment: Fresh hind tibiae were air dried for 48 h at room temperature. Air drying is one of the most common methods for drying cuticle samples. In the following, we refer to the air dried tibiae as desiccated specimens. (iv) Rehydration treatment: Desiccated hind tibiae were immerged into distilled water and kept at 4 °C for 48 h without medium.

### Mechanical testing

**Figure 1:**
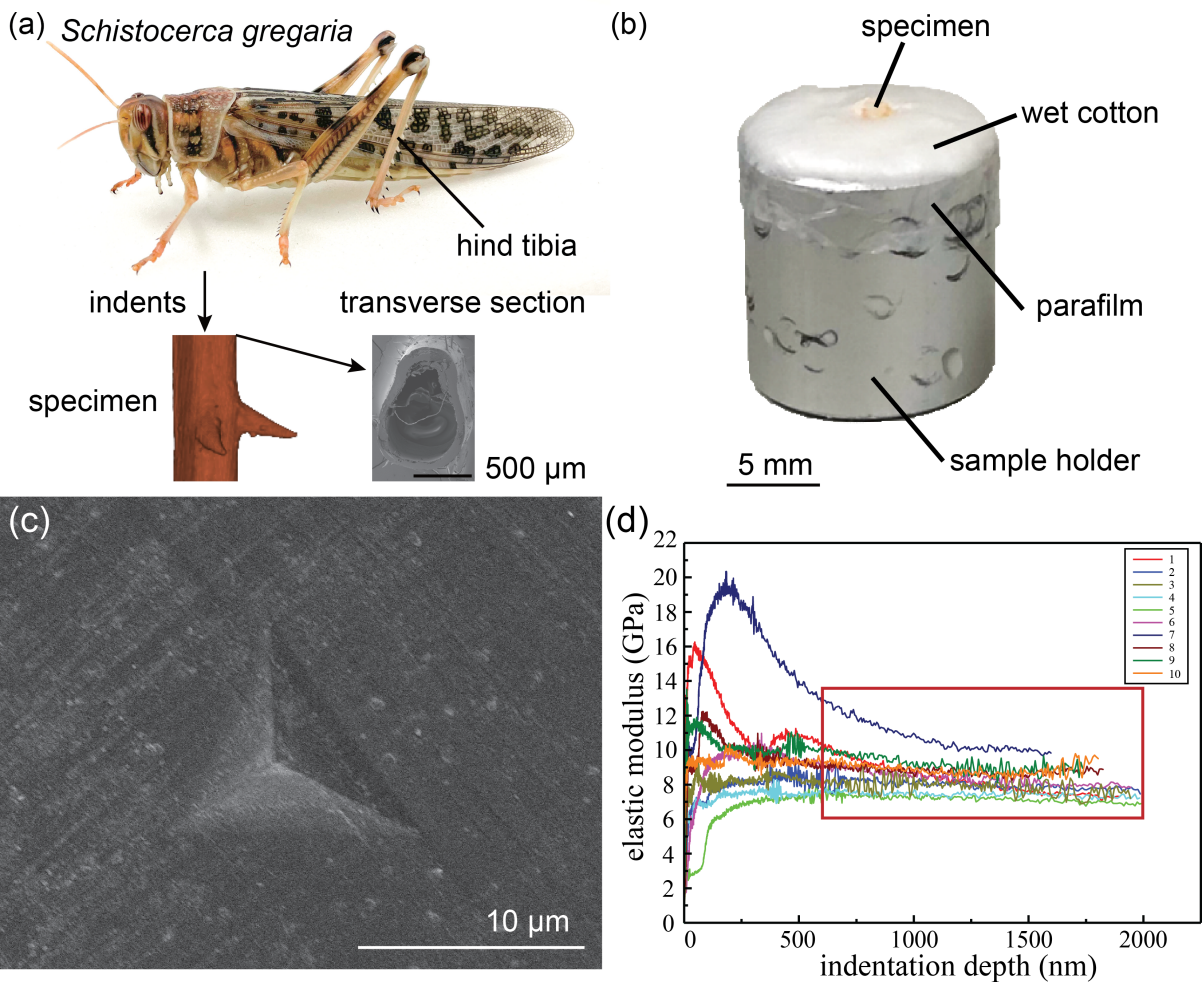
Specimens. (a) The middle part of the hind tibia was cut from the desert locust. Indents were performed on the transverse section of the specimen. (b) Tibial specimens were glued on the sample holder and surrounded by wet cotton. The wet cotton was covered by a layer of parafilm to minimize desiccation. (c) SEM image of an indent measured for a dry tibia. (d) Elastic modulus as a function of the indentation depth obtained from the CSM technique.

Pieces of about 2.0 × 0.5 × 0.5 mm^3^ size were separated from the middle part of the hind tibiae using a razor blade. They were then glued (5925 Ergo, Kisling AG, Wetzikon, Switzerland) on a sample holder within 2 min after separation from the tibiae. To avoid desiccation, except for desiccation treatment, small pieces of wet cotton were used to surround the tibiae (Figure 1b). The wet cotton pieces were covered by a layer of parafilm (BEMIS Packaging Deutschland GmbH, Rheinbach, Germany) [11]. Prior to testing, the cross section of the specimens was polished with sandpaper with a grain diameter of 0.3 µm (ITW Test & Measurement GmbH, Esslingen, Germany). We performed the indentations normal to the cross section of the tibiae. This resulted in the measurement of the elastic moduli of the tibial cuticle in the longitudinal direction (along the axis of tibia) (Figure 1).

The specimens were indented using a SA2 Nanoindenter (MTS Nano Instruments, Oak Ridge, TN, USA) equipped with a Berkovich diamond tip. The elastic modulus of the specimens was measured using the continuous stiffness measurement (CSM) technique. CSM is a well-established technique for obtaining the elastic modulus continuously as a function of the indentation depth (Figure 1d). The method involves applying a dynamic load on the top of the static load while loading. The dynamic unloading part is then used to measure the stiffness, which is further processed to calculate the elastic modulus of the cuticle [12–13]. Nanoindentations were performed on the cross sections of the tibiae on ten preset indentation sites. The distance between adjacent indentation sites was set to be larger than 30 μm, to avoid the interference between consecutive measurements. In all indentations, maximum indentation depth, strain rate, harmonic displacement, and harmonic frequency were set as 2.0 μm, 0.05 s^−1^, 1.0 nm, and 75 Hz, respectively. The Poisson’s ratio of the tibia specimens was assumed to be 0.3 [14]. The allowable drift rate was set as 0.10 nm/s to minimize the effect of vibration and thermal drift during measurements [11]. We have only used the left hind tibiae of desert locust for mechanical testing. In total, 20 left hind tibiae from 20 locust individuals underwent four treatments, namely fresh (*n* = 5), freezing (*n* = 5), desiccation (*n* = 5), and rehydration (*n* = 5). The elastic modulus reported for each indentation is the average value of the elastic moduli measured during indentations from 0.6 to 2.0 μm (red box, Figure 1d). The elastic modulus of each tibia is the average elastic modulus obtained from ten indents.

### Water content and evaporation rate measurements

To measure the water content of the specimens, paired weight measurements were performed before and after the treatments. The weight of small cut tibiae was measured twice, once at the beginning and again after 4 h (to allow for full desiccation), using an ultramicrobalance UMX2 (Sartorius Lab Instruments GmbH & Co. KG, Göttingen, Germany). In total, 30 specimens from 15 individuals were measured. The weights of 15 right hind tibiae were first measured as fresh, then after desiccation, and finally after rehydration, while 15 left hind tibiae were measured for freezing treatment. The evaporation rates of three more fresh and three more frozen specimens were measured by continuous recording of the weight of the specimens every minute for 3 h.

### Statistics

The elastic moduli and water content were compared between the different treatment groups. Significant differences were tested by one-way ANOVA tests. All statistical analyses were performed with Sigmaplot v.12.5 (Systat Software GmbH, Erkrath, Germany). All values shown in the manuscript are mean ± standard deviation (s.d.).

## Results

### Influence of sample treatment on the elastic modulus of cuticle

**Figure 2:**
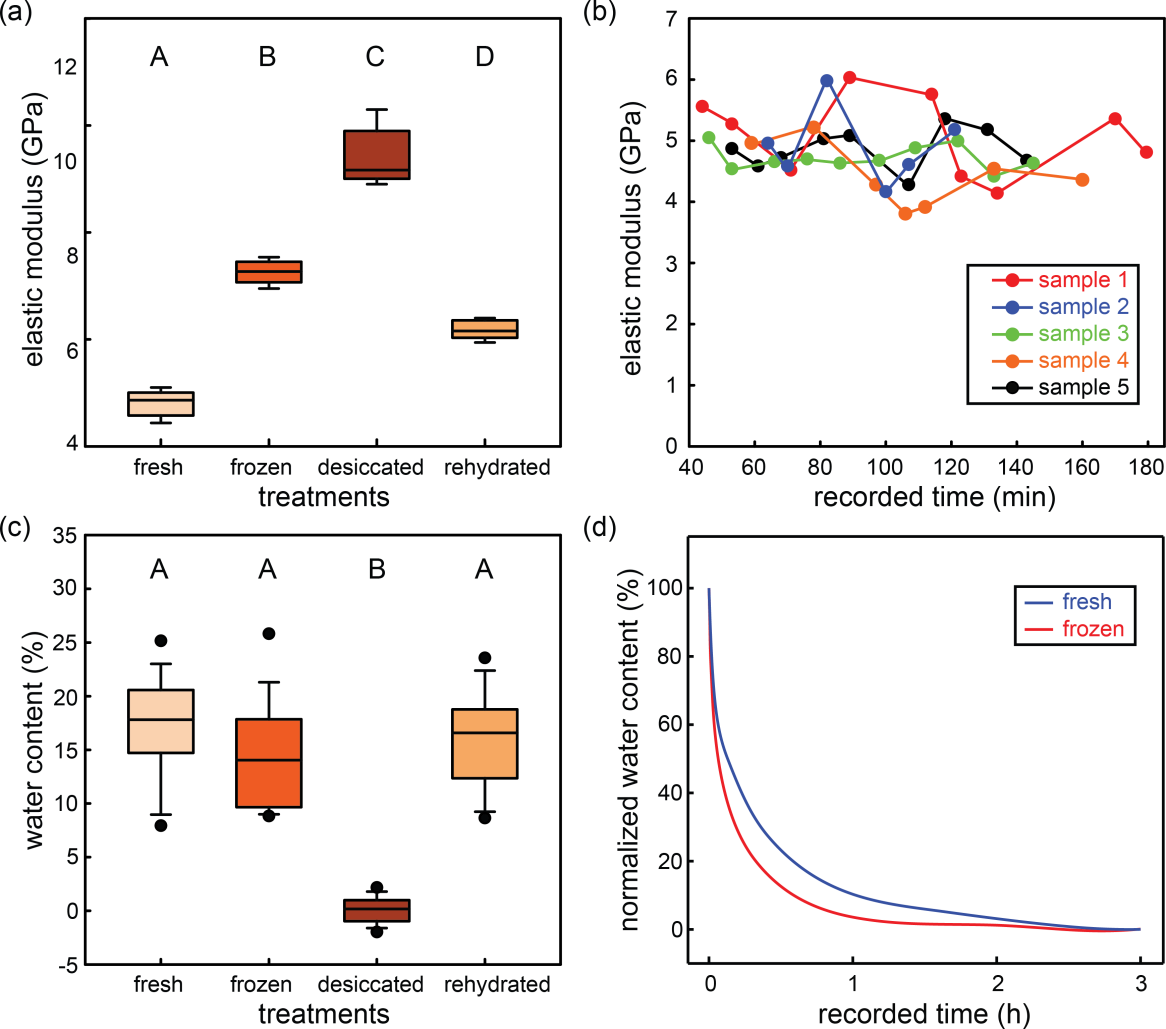
Elastic modulus and water content of specimens in different treatment groups. (a) The elastic moduli of fresh (*n* = 5), frozen (*n* = 5), desiccated (*n* = 5), and rehydrated (*n* = 5) hind tibiae are 4.8 ± 0.2, 7.3 ± 0.2, 9.4 ± 0.5, and 6.2 ± 0.2 GPa, respectively. Significant differences were determined via Holm–Sidak one-way ANOVA. Different capital letters indicate significant differences (*P* < 0.001). (b) Elastic modulus of fresh tibiae as a function of the recorded time. Different samples are presented in different colors. Each data point represents the elastic modulus obtained from a distinct indentation site. (c) Water contents of fresh (*n* = 15), frozen (*n* = 15), desiccated (*n* = 15), and rehydrated (*n* = 15) hind tibiae. Significant differences were determined via Kruskal–Wallis one-way ANOVA on ranks. Different capital letters indicate significant differences (*P* < 0.05). (d) Water contents of fresh (*n* = 3) and frozen tibiae (*n* = 3) as function of the time.

We measured the elastic moduli of hind tibiae in different treatment groups. The results obtained from nanoindentation are presented in Figure 2a. The elastic modulus values of the frozen specimens are reported from our previous study [11]. The elastic moduli of the fresh, frozen, desiccated, and rehydrated hind tibiae are 4.8 ± 0.2, 7.3 ± 0.2, 9.4 ± 0.5, and 6.2 ± 0.2 GPa, respectively. Significant differences were found between each of the two different treatment groups (Holm–Sidak one-way ANOVA, *P* < 0.001).

**Figure 3:**
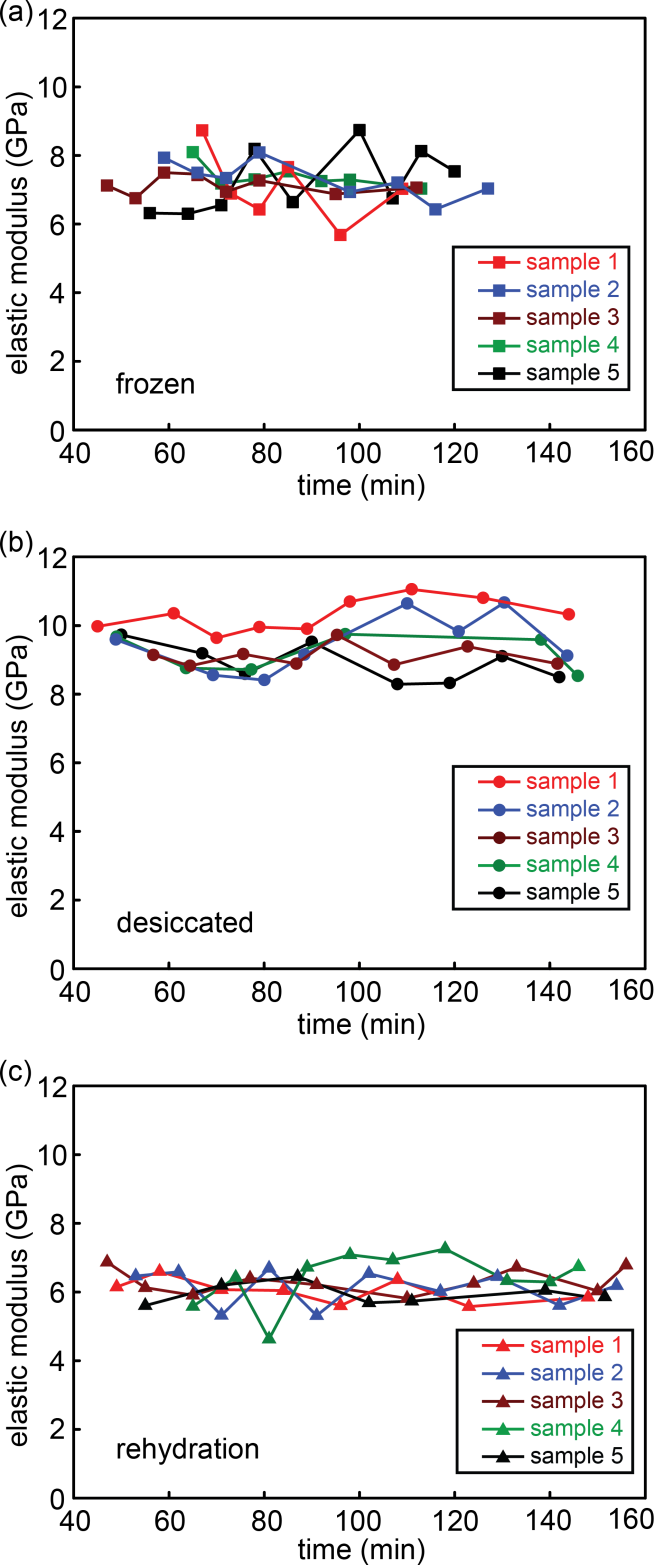
Elastic modulus of (a) frozen, (b) desiccated and (c) rehydrated tibiae against recorded time. Different colors represent different samples.

It is important to note that the results were consistent over time and no specific trend was observed in the values obtained from the measurements during the course of indentations (Figure 2b and Figure 3). In addition, the elastic moduli of the tibiae remained almost constant by increasing the indentation depth (Figure 1d).

### Water content and evaporation rates during different treatments

We measured the changes in weight of the cuticle samples caused by desiccation in the different treatment groups. This allowed us to measure the water content of the cuticle specimens. The water contents are presented by the ratio between the lost weight of a specimen and its original weight. The water content of fresh, frozen, desiccated, and rehydrated tibiae were (17.11 ± 4.98)%, (14.03 ± 4.69)%, (0.08 ± 1.16)% and (15.81 ± 4.33)%, respectively (Figure 2c). Significant differences were found between fresh/frozen/rehydrated tibiae and desiccated samples (Kruskal–Wallis one-way ANOVA on ranks, *P* < 0.05). The normalized water content of fresh and frozen tibiae as a function of the recorded time is shown in Figure 2d.

## Discussion

### Mechanical characterization of fresh cuticle

The mechanical properties of insect cuticle are strongly influenced by the level of hydration [6–8]. Hence, to obtain realistic data, performing mechanical tests on fresh cuticle samples is necessary. Giving that it is not always possible to perform mechanical measurements on living insects, two methods are frequently used to characterize the properties of “fresh” cuticle: (i) testing cuticle samples immediately after removal from living insects [5] and (ii) submerging the specimens in water before and during the test [8]. However, both of these two methods have limitations. As shown in the present study, 60% of the water content of our cuticle specimens evaporated within 15 min after removal from the insect body (Figure 2d). This can potentially lead to a significant change in the measured properties even during this short period of time. Storing the specimens in water can also cause overhydration. The increased amount of water can result in swelling of specimens, which can consequently reduce the cuticle stiffness [9,15].

Here, we suggest a new protocol that can be used to measure the elastic modulus of cuticle specimens in their native state. The level of hydration of freshly dissected cuticle specimens is maintained by surrounding them with wet pieces of cotton. In this way, the specimens can absorb water from the wet cotton pieces without being overhydrated. Our results suggest that this is a more suitable method to measure the elastic modulus of dissected cuticle specimens. We found no clear trend in the elastic modulus of our specimens prepared in this way during three hours of testing (Figure 2b and Figure 3), which additionally suggests that this preparation could maintain the water content of our specimens.

### Effect of treatments on the elastic modulus of cuticle

#### Effect of desiccation

It is well known that desiccation can change the mechanical properties, for example, stiffness and hardness, of insect cuticle. The increase in the elastic modulus, in particular, can be rather large, up to more than tenfold of the stiffness of cuticle in the native state [16]. This is because water makes cuticle soft and compliant. A significant increase was also observed in the elastic modulus of our specimens after desiccation. The elastic modulus of the desiccated specimens was about twice that of the fresh specimens. This relatively small increase in the elastic modulus observed here is likely due to a higher level of sclerotization of our cuticle specimens in comparison to the previously tested ones. A highly sclerotized cuticle sample contains less water in comparison to a less sclerotized sample. Thereby, desiccation would have a lesser effect on the properties of sclerotized samples.

#### Effect of rehydration

In a previous study, Klocke and Schmitz [8] measured the elastic modulus of rehydrated specimens of locust sternal cuticle. They found that the elastic moduli obtained from their experiments were higher than those reported in the literature for similar cuticle parts. They, therefore, suggested that rewetting of a cuticle specimen might not return its hydration level to the native state. Our results also showed that the elastic modulus of rehydrated cuticle sample is significantly higher than that of fresh specimens. However, surprisingly, we found no significant difference between the water content of the fresh and rehydrated cuticle samples (Figure 2c). This raises the question about the source of the observed difference between the elastic moduli of fresh and rehydrated cuticles. It might be due to the changes in the interaction of water molecules with the chitin of the cuticle after rehydration. Insect cuticle is a biological composite with a protein matrix and embedded chitin fibers [17]. It is the only biological composite that has been modelled successfully using the modified Voigt estimate of the composite elastic modulus [15]. According to the Voigt model, the effective elastic modulus parallel to the fibers in a unidirectional fiber composite, such as locust hind tibial cuticle [11], can be estimated by [15]


[1] $$E=E_{\text{f}}V_{\text{f}}+E_{\text{m}}V_{\text{m}},$$

where *E*_f_ and *V*_f_ are, respectively, elastic modulus and volume fraction of the fibers, and *E*_m_ and *V*_m_ are elastic modulus and volume fraction of the matrix, respectively.

Most of the water in the cuticle is associated with the protein matrix, where free water can be added to or taken from [1]. Thus, the elastic modulus of the matrix might not show a difference between the fresh and rehydrated states. The elastic modulus of fibers, however, is affected by the density of effective hydrogen bonds (H-bonds) [18]. Addition or removal of water can presumably disrupt these structural H-bonds and, therefore, affect the elastic modulus of cuticle [15]. This effect, based on the results of our study and those previously reported by Klocke and Schmitz [8], is likely to be irreversible.

#### Effect of freezing

According to our results, the elastic modulus of frozen tibia is significantly higher than that of fresh and rehydrated cuticle. This is in contradiction to the previously reported data obtained from three-point bending tests [9], which showed no significant difference between the elastic moduli of fresh and frozen tibiae. This contradiction is probably due to one key reason, namely the different desiccation rates of specimens prepared for nanoindentation and bending tests. In fact, desiccation of natively hydrated cuticle samples is an inevitable process if they are not continuously supplied by hemolymph. The desiccation rate is faster near cuticle surfaces. Hence, it is likely that small nanoindentation specimens are more prone to desiccation than larger bending tests specimens, which have smaller surface areas with respect to their total volume. It must be clarified whether the desiccation discussed here happens during the nanoindentation measurement instead of during the freezing process.

#### Water content of locust tibial cuticle

According to previous reports, the water content of cuticle varies between 12% and 75%, depending on the sclerotization level [1]. Our results indicated a water content of ca. 17%, which is in agreement with literature data. Nevertheless, previous studies on locust hind tibia showed that the water content of this body part can be up to 47% [5,9]. We expect this difference is due to the influence of hydrated soft tissues (e.g., membranes and muscles) that are attached to the interior wall of the tibial cuticle. In our study, we carefully removed the inner tissues, whereas the previous studies have been performed on the whole tibia.

## Conclusion

The complex relationship between multiple factors, such as microstructure, material composition, and sclerotization, has provided cuticle with properties that are unique to this biological material. Understanding the mechanisms behind the remarkable properties of cuticle can inspire the design of engineering composites with enhanced properties. Specifically, cuticle can help us to develop ceramics with improved fracture toughness or overcome limitations of existing engineering materials by combining conflicting properties. A first step towards these aims is to develop guidelines for measuring cuticle properties accurately.

Our results show that none of the examined treatment methods, that is, freezing, desiccation, and rehydration, can represent the elastic modulus of native cuticle specimens when tested by nanoindentation. Considering that no significant difference was found between the water content of the fresh, frozen, and rehydrated specimens, the difference in their elastic moduli cannot be solely attributed to the presence/absence of water. Instead, other additional factors, such as desiccation rate and/or how the water molecules interact with other molecules within the cuticle, are important. Our study can be used as a template for future studies that aim to measure cuticle properties using nanoindentation or other nano- or micromechanical testing methods.

## Data Accessibility

All supporting data are made available in the article.

## Authors’ Contributions

Conceptualization: SNG and HR; formal analysis: CL; funding acquisition: CL and SNG; investigation: CL; methodology: CL, HR, and SNG; resources: SNG; supervision: HR and SNG; validation: CL; visualization: CL; writing – original draft preparation: CL and HR; writing – review and editing: CL, HR and SNG.
